# Ultrasound-targeted microbubble destruction mediated herpes simplex virus-thymidine kinase gene treats hepatoma in mice

**DOI:** 10.1186/1756-9966-29-170

**Published:** 2010-12-23

**Authors:** Shiji Zhou, Shengwei Li, Zuojin Liu, Yong Tang, Zhigang Wang, Jianping Gong, Changan Liu

**Affiliations:** 1 Department of Hepatobiliary Surgery, the Second Affiliated Hospital of Chongqing Medical University, 76 Linjiang Road, Yuzhong District, Chongqing 400010, PR China; 2 Institutional of Ultrasound Imaging, the Second Affiliated Hospital of Chongqing Medical University, 76 Linjiang Road, Yuzhong District, Chongqing 400010, PR China

## Abstract

**Objective:**

The purpose of the study was to explore the anti-tumor effect of ultrasound -targeted microbubble destruction mediated herpes simplex virus thymidine kinase (HSV-TK) suicide gene system on mice hepatoma.

**Methods:**

Forty mice were randomly divided into four groups after the models of subcutaneous transplantation tumors were estabilished: (1) PBS; (2) HSV-TK (3) HSV-TK+ ultrasound (HSV-TK+US); (4) HSV-TK+ultrasound+microbubbles (HSV-TK+US+MB). The TK protein expression in liver cancer was detected by western-blot. Applying TUNEL staining detected tumor cell apoptosis. At last, the inhibition rates and survival time of the animals were compared among all groups.

**Results:**

The TK protein expression of HSV-TK+MB+US group in tumor-bearing mice tissues were significantly higher than those in other groups. The tumor inhibitory effect of ultrasound-targeted microbubble destruction mediated HSV-TK on mice transplantable tumor was significantly higher than those in other groups (p < 0.05), and can significantly improve the survival time of tumor-bearing mice.

**Conclusion:**

Ultrasound-targeted microbubble destruction can effectively transfect HSV-TK gene into target tissues and play a significant inhibition effect on tumors, which provides a new strategy for gene therapy in liver cancer.

## Introduction

Hepatocellular carcinoma (HCC) is one of the malignant tumors with high incidence around the world [1,2]. More than one million new cases appeared each year, particularly in the Asia-Pacific region. This disease has rapid progress, high recurrence rate and traditional treatments have limited. With the continuous development of molecular biology, gene therapy for liver cancer has become a research hotspot and direction [3]. However, the safety of viral vector, ineffectiveness of non-viral gene vectors and other problems limit its further development [4,5]. Therefore, the search for an efficient, well targeting and safe gene transfection system for cancer gene therapy has become a focus of reseachers inteset.

Recently a large number of studies have shown that ultrasound-targeted microbubble destruction is a safe, effective, non-invasive, and physical gene transfection technology, which brings a new hope for gene therapy in liver cancer [6-8]. Ultrasound microbubbles mostly contain gas [9]. The composition of its shell may include albumin, lipids, saccharide, non-ionic surfactants, polymers and other materials [10]. At present the size has been developed to nano-scale and it has the ability to penetrate the vascular endothelium [11]. Microbubbles containing gas will be compressed and expansed under the action of ultrasound with a certain intensity and frequency. When the sound energy reaches certain intensity, the microbubbles are immediately crushed. This will produce cavitation effect and mechanical effect to increase the permeability of cell membrane structure in target region, make the microvessels with the diameter ≤7 μm break down, widen the intercellular gap of vascular endothelial cells. The exogenous genes can easily penetrate into the tissues and cells through capillary vessels to improve the gene transfection rate and expression [12,13]. Cavitation effect can also damage cells, inhibit cell proliferation, and promote tumor cell apoptosis. When ultrasound-targeted microbubble generates strong cavitation effects, it can also damage blood vessel wall, active endogenous or exogenous coagulation, induce large-scale capillary embolism and block nutrient supply to cancerous cells, leading to disappearance of tumor tissues [14,15].

Suicide gene therapy has been widely used in liver cancer treatment and showed a good application prospect. Especially the herpes simplex virus thymus kinase/ganciclovir (HSV-TK/GCV) therapy system is most widely applied. HSV-TK is a prodrug enzyme gene which can express and produce TK in the tumor cells, catalyze nucleoside analogue to form mono- phosphate products, and further form a triphosphoric acid product under the effect of phosphokinase in the cell. As a chain terminator, it will interfere with DNA synthesis during cell division, leading to tumor cell death [16,17]. A large number of studies have shown that suicide gene system also has a "bystander effect". The effect will kill non-transfected cells with the transfected cells, which overcomes the shortcomings of the low gene transtection rate and greatly enhances the anti-tumor effect of suicide gene therapy [18].

In this study, ultrasound microbubbles wrapped HSV-TK suicide gene had targeted release in mice liver tissues, and improved gene transfection efficiency with the features of ultrasound and microbubbles. In addition, the bystander effect of suicide gene fully played the anti-tumor role. The study provided an efficient, relatively targeted, non-invasive, and physical gene transfection method for HSV-TK/GCV system.

## Materials and methods

### Preparation of lipid microbubbles

Dipalmitoyl phosphatidylcholine (DPPC) (sigma, USA), distearoyl phosphatidyl ethanolamine (DSPE) (sigma, USA), diphenyl phosphoryl azide (DPPA) (sigma, USA), glycerol, PBS were mixed according to a certain proportion and were placed in a 1.5 ml vial, The vials were incubated at 40°C for 30 minutes. Each vial was filled with the perfluoropropane gas (C3F8), then the vials were mechanically shaken for 45 seconds in a dental amalgamator (YJT, Shanghai Medical Instrument Co., Ltd.) and quiescence for 5 min. This solution was diluted by phosphate-bufferedsaline, sterilized by Co60 and stored at 4°C;. Then the self-made lipid microbubbles were made. The average diameter was 1.82 ± 0.45 μm; the average concentration was 1.2 × 10^10^/ml; the average potential was -24.7 ± 0.56 mV (n = 4).

### Plasmid

The pORF-HSVTK plasmid was carried out PCR amplification with upstream primer TKF(ACGCGTCGACATGGCCTCGTACCCCGGCCATCAACAC) and downstream primer TKR (CGCGGATCCTCAGTTAGCCTCCCCCATCTCCCGGG) to obtain about 1.2 kb target HSV-TK fragment. Then directionally connect HSV-TK target gene fragment and pIRES2-EGFP (Invitrogen, USA) vect with the help of DNA ligase to obtain recombinant plasmid pIRES2-EGFP-TK. The recombinant plasmid was transformed into DH5a Escherichia coli competent cells and spread on onkanamycin resistant LA plate for culture of 12-16 h. When the colonies grew out, we selected positive clones to extract plasmid, followed by Sal I and BamH I enzymes cut identification and sequencing by TaKaRa Company.

### Connection of microbubbles with plasmid

According to the method of preparation of gene-loaded lipid microbubbles from the reference of Zhaoxia Wang [19]. We mixed the prepared blank lipid microbubbles and poly-L-lysine (1 mg/ml) (Sigma Corporation, USA), and cultured at 37°C; for 30 min. Subnatant was soaked and deserted and washed twice by PBS. Naked plasmid (1 mg/ml) was added and incubated at 37°C; for 30 min, and washed by PBS twice. The manipulation was repeated three times. then gene-loaded lipid microbubbles were made. It was measured the average diameter of the HSV-TK wrapped microbubbles was between 2 μm to 4 μm and the concentration was 6.9 × 10^9^/ml. The potential was -3.7 ± 0.56 mv (n = 4) and the plasmid concentration was 0.1 μg/μl.

### Animal model

The study protocol was approved by the Animal Research Committee of our institution.40 Kunming mice, cleaning grade, body weight (20 ± 2 g), male, 6 to 8 weeks old, were purchased from the Laboratory Animal Center of Third Military Medical University. H22 tumor cells (from Institute of ultrasonography, the second affiliated Hospital of Chongqing Medical University as a gift) were cultured in the RPMI 1640 medium (Hyclone, China) containing 10% betal bovine serum (FBS) at 37°C; with 5% CO2. We used serum-free RPMI1640 medium to adjust cell concentration to about 1 × 10^7^/ml, followed by placenta blue exclusion dye test. The detected cell activity was >90%. Each mouse was inoculated 0.2 ml cell suspension subcutaneously in the right flank of Kunming mice. The tumor diameter was 0.5-1.0 cm after one week with the tumor rate of 100%. Experimental animals were randomly divided into four groups (10/per group) :(1) PBS group; (2) HSV-TK group; (3) HSV-TK+ US group; (4) HSV-TK+MB+US group.

### In vivo transfection by ultrasound combined with HSV-TK gene microbubbles

The microbubbles containing HSV-TK plasmid were injected through the tail vein of mice, 200 μl for each time. The mice were injected once every 3d and consecutively injected for 3 times. Group A: PBS (200 μl); Group B: HSV-TK (200 μl, 0.1 μg/μl); group C: US+HSV-TK (200 μl, 0.1 μg/μl); Group D: US+HSV-TK+MBs (200 μl, 0.1 μg/μl). Self-made ultrasonic gene transfection instrument (UTG 1025, Institute of Ultrasound Imaging of Chongqing Medical Sciences, Chongqing, China) was applied on C and D groups for irradiation after the target gene injection, with the radiation frequency of 1 MHz, sound intensity of 2 W/cm^2^, and used pulse irradiation method for 5 min, with the interval time of 10 s. Each mouse was intraperitoneally injected 0.2 ml (100 mg·kg^-1^·d^-1^) GCV (Roche, Switzerland) 48 h after irradiation, which last for 14 days.

### Western-blot

Proteins were extracted using protein extraction reagent,48 hours after transfection and save at -20°C;, following a protocol provided by the manufacture. TK protein expression was detected with western-blot. 40 ml/L concentrated gel, 100 ml/L separation gel, pre-stained protein Marker 3.0 μL, 20 μg/hole sample total protein. Add sample into 100 mL/L SDS-PAGE followed by electrophoresis at 60 V. Change voltage to 100 V after 30 min. Get the gel when bromophenol blue ran to the bottom after 90 min. Synchronously transfer the protein to PVDF membrane at 20 V for 50 min. Seal for 4 h with 50 mL/L skim milk TBST at room temperature after trarsmembrane; add primary antibody (TK1 Polyclonal antibody, 1:500) (Abcam, United Kingdom) followed by incubation for 2 h at room temperature and staying overnight at 4°C;. Use TBST to wash membrane three times with 15 min/time. Add appropriate concentration of secondary antibody combined with HRP (1:5000) for incubation followed by jiggle at room temperature for 2 h, washing membrane, imaging and exposure. The protein bands were normalized with β-actin, and all blots were quantified with Software Quantity One (Bio Rad).

### Detection of tumor cell apoptosis with TUNEL staining

After the treatment, the tumor tissues were routinely paraffin-embedded and made into 5 μm slices. The sections were dewaxed with xylene followed by gradient alcohol hydration. Add 20 μg/ml free-DNase protease K and keep at 37°C; for 15 minutes. Then wash three times with PBS followed by incubation in 3% hydrogen peroxide (H_2_O_2_) at room temperature for 10 minutes. Then wash three times with PBS. Add 10 μl b-11-DUTP and 10 μL TDT to 1 ml Tunel buffer followed by reaction at 37°C; for 1 h and at room temperature for 1 h; Streptavidin-HRP (1:400) reaction for 30 min; 0.04% DAB+0.03% H_2_O_2 _color development for 10 min; hematoxylin contrast dye, differentiation with hydrochloric acid and ethanol, washing, and sealing with conventional resin. Then under the optical microscope with 400 times magnification, five tumor cell areas were randomly selected. Count the number of total cells and apoptotic cells to calculate the percentage of TUNEL staining positive cells, i.e., apoptotic index (AI). AI = (number of apoptotic cells/the total number of tumor cells) × 100%.

### Assessment of therapeutic effect

Measure the tumor size regularly to calculate the inhibition rate: during treatment use calipers to measure the maximum diameter a (cm) and the shortest diameter b (cm) of tumors every 3 d, and apply the formula V = ab^2^/2 to calculate the tumor volume with the unit of cm^3^. The tumor inhibition rate = (the average size of tumors in control group- mean tumor volume in treatment group)/mean tumor volume in control group × 100%. According to the size of the measured tumor volume, draw the growth curves. Take five mice in each group for the observations of survival time. The observation lasts for 80 days and survival curves were drawn.

### Statistic analysis

The SPSS17.0 statistic software was used to make a statistic analysis. The measurement data was expressed as mean ± SD. The analysis of variance was used to assess the inhibition rate. LSD-t test was used for pairwise comparison. Kaplan-Meier method was applied for survival analysis. A *P *value less than .05 was considered indicative of a statistically significant difference.

## Results

### HSV-TK in vivo transfection effect

**Figure 1:**
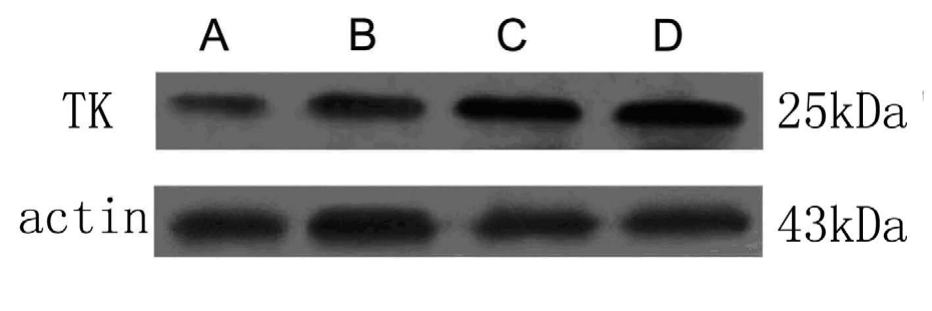
**The expression of TK protein was detected by Western-blot 48 h after transfection**. Each group has a single band at 25 kDa and the TK protein expression was the highest in the HSV-TK+ US+MB group (A. PBS group; B. HSV-TK; C. HSV-TK+US; D. HSV-TK+US+MB).

48 h after the transfection of ultrasound microbubble mediated HSV-TK in mice, the TK protein expression was detected in tissues by western-blot. It was observed that a single band appeared in each group at 25 kd. The band in HSV-TK+US+MBs group was the most obvious (Figure 1).

### Apoptosis

**Figure 2:**
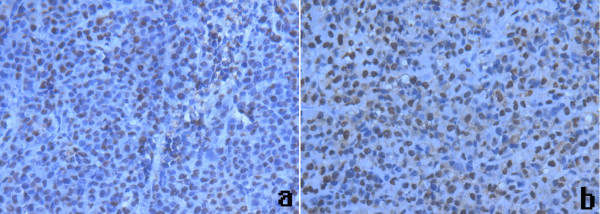
**Apoptosis expression in four groups of mice liver cancer tissues (original magification × 400)**. Terminal deoxyuridine nick end-labeling results showed that cells stained brown in nuclei were apoptotic cells. The tumor cells in two groups appear apoptosis in varying degree. (a. HSV-TK+US group, b. HSV-TK+US+MB).

In order to further confirm that microbubble mediated HSV-TK/GCV treatment system can induce apoptosis of tumor cells. We applied TUNEL staining to detect tumor cell apoptosis in each group. When cells underwent apoptosis, DNA double-strand broke and dUTP could be marked at the DNA breakage. As can be seen from each group, the tumor cells in each group appeared apoptosis in different degrees. The tumor cell apoptosis in HSV-TK+US+MBs+ GCV group was the most obvious (Figure 2). Apoptotic index comparison: group D vs group C, P < 0.05; group D vs group A, P < 0.001; group A vs group B, P > 0.05 (Table 1).

**Table 1:** The apoptotic index of tumor tissues in each group ( ± s)

Group	PBS group	HSV1-TK group	HSV1-TK+US	HSV1-TK+US+MB
AI(%)	12.1 ± 2.0	16.8 ± 2.3	23.5 ± 3.1^#^	38 ± 3.6*^#^

Compared with control group, #p < 0.05; compared with other groups, *p < 0.001

### Treatment effect

**Figure 3:**
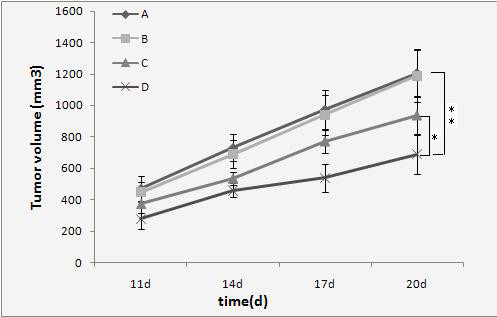
**It can be seen from the tumor growth curve that the tumor growth in HSV-TK+US+MB group was significantly inhibited**. Compared with control group, **P < 0.01; compared with HSV-TK+US group, *P < 0.05.A. PBS; B. HSV-TK; C. HSV-TK+US; D. HSV-TK+US+ MB).

**Figure 4:**
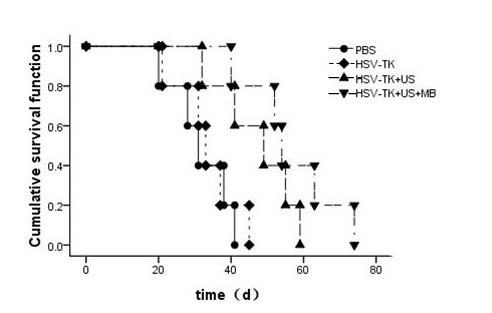
**The survival time of five tumor-bearing mice in each group is observed for 80 days**. It can be seen from the survival curves of tumor-bearing mice that the survival time of tumor-bearing mice in HSV-TK+US+MB group is significantly prolonged.

As the tumor increases, the mice show obviously emaciated body, appetite loss, dull furs, activity reduction, body weight loss and so on. However, after treatment the mice growth in the GCV treatment group is significantly better than the control group. It can be seen from the tumor growth curve (Figure 3) that the tumor growth in group D (HSV-TK+US+MB) slows down significantly. Compared with the tumor size of control group A (PBS), the tumor sizes of group D were smaller than group A at all time points with statistical significance (P < 0.01). The tumor inhibition rates of group A, B, C and D were: 0%, 3.90% ± 1.80%, 22.70% ± 2.86% and 41.25% ± 3.20%. Take five mice tumor-bearing in each group as an 80-day continuous observation of their survival time. It can be seen from the survival curves (Figure 4) that group D has a significant difference (P < 0.05) with other groups in improving the survival time of tumor-bearing mice.

## Discussion

Liver cancer gene therapy requires a non-invasive, efficient, targeting and safe gene transfection technology. However, ultrasound-targeted microbubble destruction technology provides a good physical gene transfection method. The ultrasound can be applied to monitor and crush the microbubbles in target tissues at the specific time and space to achieve the accuracy and targeting for gene therapy. The cavitation and mechanical effects generated by ultrasound-targeted microbubble destruction can increase membrane permeability in target areas and widen the gap of vascular endothelial cells, making it easier for foreign gene into the target tissue. Most studies have indicated that under certain ultrasonic irradiation conditions, ultrasound did not destroy the transfection gene, but enhanced the transfection efficiency of target genes [20,21].

In this study, microbubble wrapped HSV-TK plasmid was intravenously injected into mice, followed by ultrasound irradiation to tumors in order to smash the microbubbles for the targeted release of HSV-TK gene. 48 h after transfection, TK protein expression in HSV-TK+ US+MB+GCV (group D) was significantly higher. The valid expression of TK protein in the target area is the premise for tumor treatment HSV-TK/GCV. From the final treatment effect, the anti-tumor effect of HSV-TK+US+MB group was the highest amony other groups, and the survival time of tumor-bearing mice could be prolonged.

At present, most of the studies in which microbubbles were chosen as gene carriers applied the method of mixture of microbubble and gene for transfection [22]. Using this approach for gene transfection may affect the foreign gene transfection efficiency in the target tissues, making the targeted expression of foreign gene decrease. In this study, the method of preparation of microbubble from Wang et al was selected [19]. Through the principle of electrostatic adsorption, the target genes become a part of the microbubble shells. This will not only increase the amount of gene carried by microbubbles, but also make use of microbubble shells to prevent the foreign gene from being degraded by DNA enzymes in the blood. Thereby target gene expression in the target tissue was increased.

Ultrasound-targeted microbubble destruction technology for gene transfection is a kind of transient transfection. Gene expression time in organizations is relatively short, rather than other virus-mediated foreign gene expressions for sustainable long time. The studies from Aoi A et al have shown that in this method target gene will obviously decreased 48 h after transfection, which may be related to the rapid degradation after plasmid DNA transfection [23]. In this study, the method of multiple dosing of HSV-TK gene was applied to overcome the shortcoming that exogenous genes can not constantly express in transient transfection. The method of multiple dosing of target gene also shows a great help for the treatment of tumor. At the same time a lot of studies have shown that microbubble is a safe, reusable carrier which will cause immune response rarely which provides an evidence for multiple dosing of gene in this study [24].

HSV-TK suicide gene in this study is a pro-drug enzyme gene. It can transform non-toxic pro-drugs GCV into cytotoxic drugs by phosphorylation to play an anti-tumor effect. The TK gene will cause tumor cell death ultimately with the process of apoptosis [25]. We used TUNEL staining to assess the tumor apoptosis in all groups. Compared with the control group, the tumor cell apoptosis in US+HSV-TK group and HSV-TK+US+MB group was more obvious. The apoptosis index of HSV-TK+US+MB group was the highest in the four groups. This phenomenon illustrates that the microbubble wrapped HSV-TK can significantly increase the TK gene transfection under the ultrasonic irradiation and enhance the anti-tumor effects of HSV-TK/GCV system. On the other hand, the bystander effect of HSV-TK/GCV system is also strong. Those cells which have not been transfected can be supplemented by "bystander effect" to play a good anti-tumor effect [26].

In conclusion, we used an ultrasound contrast agent as a new type of gene delivery vector, and the anti-tumor efficacy of HSV-TK was markedly improved. Ultrasound- targeted microbubble destruction technology is expected to become a new gene delivery means and may provide a novel strategy for targeted cancer therapy.

## Competing interests

The authors declare that they have no competing interests.

## Authors' contributions

SZ, JG, CL participated in the experiments design of the study and coordination. The plasmidpIRES2-EGFP -TK is constructed by SZ and YT. H22 cells and cultivation is finished by SZ. Experimental of mice model finished by SZ and SL. Apoptosis and Western-blot is finished by SZ and ZL. SZ and ZL participated in the performed the statistical analysis. SZ and ZW participated in the preparation of lipid microbubbles. All authors read and approved the final manuscript.
